# “This is an illness. No one is supposed to be treated badly”: Community-based stigma assessments in South Africa to inform TB stigma intervention design

**DOI:** 10.21203/rs.3.rs-3716733/v1

**Published:** 2023-12-11

**Authors:** Isabel Foster, Amanda Biewer, Nosivuyile Vanqa, Goodman Makanda, Phumeza Tisile, Sally E. Hayward, Dillon T. Wademan, Michaile G. Anthony, Rachel Mbuyamba, Michelle Galloway, Wieda Human, Helene-Mari Westhuizen, Jon S. Friedland, Andrew Marino-Medina, Ingrid Schoeman, Graeme Hoddinott, Ruvandhi R. Nathavitharana

**Affiliations:** Oxford University; Beth Israel Deaconess Medical Center, Harvard Medical School; Desmond Tutu TB Centre, Department of Paediatrics and Child Health, Faculty of Medicine and Health Sciences, Stellenbosch University, South Africa; TB Proof; TB Proof; University of London; Desmond Tutu TB Centre, Department of Paediatrics and Child Health, Faculty of Medicine and Health Sciences, Stellenbosch University, South Africa; Desmond Tutu TB Centre, Department of Paediatrics and Child Health, Faculty of Medicine and Health Sciences, Stellenbosch University, South Africa; TB Proof; TB Proof; TB Proof; Oxford University; University of London; Desmond Tutu Health Foundation; TB Proof; Desmond Tutu TB Centre, Department of Paediatrics and Child Health, Faculty of Medicine and Health Sciences, Stellenbosch University, South Africa; Beth Israel Deaconess Medical Center, Harvard Medical School

**Keywords:** Tuberculosis, Stigma, Intervention, Cascade of care, Community-engaged research

## Abstract

**Background:**

Though TB-related stigma is a recognized barrier to care, interventions are lacking and gaps remain in understanding the drivers and experiences of TB-related stigma. We undertook community-based mixed methods stigma assessments to inform stigma intervention design.

**Methods:**

We adapted the Stop TB Partnership stigma assessment tool, and trained three peer research associates (PRAs; two TB survivors, one community health worker) to conduct surveys with people with TB (PWTB, n=93) and caregivers of children with TB (n=24) at peri-urban and rural clinic sites in Khayelitsha, Western Cape, and Hammanskraal, Gauteng Province, South Africa. We descriptively analyzed responses for each stigma experience (anticipated, internal, and enacted), calculated stigma scores, and undertook generalized linear regression analysis. We further conducted 25 in-depth interviews with PWTB (n=22) and caregivers TB (n=3). Using inductive thematic analysis, we performed open coding to identify emergent themes, and selective coding to identify relevant quotes. Themes were organised using the CARD (Constraints, Actions, Risks and Desires) framework.

**Results:**

Surveys revealed at least one-third of PWTB and one-quarter of caregivers report experiences of anticipated, internal, and/or enacted stigma, which affected engagement throughout the care cascade. Participants in rural locations (compared to peri-urban) reported higher anticipated, internal, and enacted stigma (β-coefficient 0.72, 0.71, and 0.74). Interview participants described how stigma experiences, including HIV intersectional stigma, act individually and in concert as key constraints to impede care, and underpins failure to disclose a TB diagnosis, isolation, and exclusion. Stigma resilience arose through understanding that TB can affect anyone and should not diminish self-worth. Risks of stigma, driven by fears related to disease severity and infectiousness, led to care disengagement and impaired psychological wellbeing. Participants desired counselling, identifying a specific role for TB survivors as peer counsellors, and community education.

**Conclusions:**

Stigma is highly prevalent and negatively impacts TB care and the well-being of PWTB, warranting its assessment as a primary outcome indicator rather than intermediary contributor to poor cascade outcomes. Multicomponent stigma interventions are needed, including counselling for PWTB and education for health workers and communities. Such interventions must incorporate contextual differences based on gender or setting, and use survivor-guided messaging to foster stigma resilience.

## BACKGROUND

Tuberculosis (TB) remains a leading cause of death globally and in South Africa (1). TB stigma is a recognized barrier to care, causing delays in care-seeking and treatment initiation, and gaps in engagement and adherence (2). Stigma manifests in different forms including enacted stigma through the negative actions of others, anticipated stigma based on the expected negative actions of others, and internal stigma arising from negative actions or beliefs towards one’s self (3–5). Strategic documents such as the World Health Organization (WHO) End TB strategy and United Nations High-Level Meeting on TB include stigma reduction as an important strategy to decrease TB transmission and disease burden (6, 7). Yet, evidence to inform the design and implementation of TB stigma interventions is limited (8).

Despite clear guidance on TB stigma measurement (9), existing stigma intervention studies use heterogeneous approaches to define and measure stigma experiences, and most do not have stigma reduction as a primary outcome (8). An exploratory analysis of TB stigma within a larger contact tracing study in South Africa illustrates how individuals with TB are marked by TB stigma, resulting in reluctance to engage with both facility and home-based services (10). Other data from South Africa highlight how TB care engagement is affected by factors such as gender and community social standing, both of which are linked to stigma (11). There remain major gaps in our understanding of TB stigma, its drivers, its differential effects on groups based on gender or setting, and its impact on TB outcomes. These gaps impede the design of interventions to address stigma and improve TB outcomes.

The cascade of care model quantifies gaps in the retention of people with TB at each stage of care from care-seeking to diagnosis to treatment and cure, and has shown that only about 50% are estimated to complete treatment (12). A care cascade analysis for South Africa reported that 25% of the estimated 532 005 people with TB are lost between accessing TB diagnostic testing and initiating treatment, and 19% are lost between treatment initiation and completion (13), underlining the need to improve care engagement. Human Immunodeficiency Virus (HIV) research has shown that stigma is not experienced uniformly across the care cascade (14), highlighting that the efficacy of stigma interventions relies on addressing the type of stigma being experienced (internal, anticipated or external). Understanding how TB stigma manifests according to stages of care, is essential to design effective, evidence-based interventions.

Mixed methods approaches can provide a fuller understanding of complex care engagement and how this maps to measurable programme targets than qualitative or quantitative data alone (15, 16). In recent years, there has been a growing use of community-engaged research to enable meaningful and equitable research participation by affected communities (16). The use of peer research associates (PRAs), who have lived experiences of the diseases being studied, has been successfully implemented for diseases such as HIV and cancer (17, 18). However, PRAs remain an underexplored opportunity for people affected by TB to engage with, inform and contribute to TB research. With the overarching goal of designing and implementing a contextually relevant stigma reduction intervention, we employed a community-based participatory mixed methods research approach, co-led by TB survivor PRAs, to understand how, when, and where stigma is experienced by people affected by TB.

## METHODS

### Study design and setting

We conducted a mixed methods study with a convergent design (19). From January 2021 to March 2022 we undertook community-based stigma surveys and in-depth interviews with people affected by TB. Participants were recruited from two communities located around purposively selected clinic sites in geographically distant provinces in South Africa: (1) Luvuyo Clinic in Khayelitsha, City of Cape Town Health District, Western Cape Province, and (2) Hammanskraal Clinic in Hammanskraal, Tshwane Health District, Gauteng Province. These sites were chosen based on TB incidence and representation of peoples from two major ethnic groups (isiXhosa and Sotho) in a peri-urban and rural setting. In 2019, TB incidence was 527 per 100 000 population in the City of Cape Town district and 181 per 100 000 population in the Tshwane District (20). This community-based participatory research project engaged persons with lived experience of TB as PRAs following the framework of Kaida et al. (21). PRA training was provided using a hybrid learning environment including remote training sessions and in-person coaching from experienced qualitative researchers (GH, NV, DTW). PRAs (GM and PT) were involved at all stages from project conception to analysis, through dissemination of findings.

### Study population, and sampling recruitment

We sought to recruit people who were aged ≥ 18 years and defined as 1) PWTB based on a current or prior diagnosis of TB, or 2) caregivers who had cared for a child (< 18 years) with TB. Survey participants were identified by community health workers (CHWs) working at either of the selected study clinic sites, and recruited using convenience sampling. From the participants who consented for and completed the quantitative survey, we purposively selected participants for in-depth interviews. Purposive sampling sought to ensure representation of: 1) people with TB (current or previous), 2) caregivers, 3) gender, 4) TB type (drug susceptible or resistant; pulmonary or extra-pulmonary) and 5) HIV status.

### Stigma measurement tools

We adapted the validated Stop TB Partnership TB Stigma Assessment Tool (22) based on literature review and iterative feedback from our PRAs who pre-tested the survey. The same questions were asked of PWTB and caregivers, with questions for the caregivers focusing on whether they thought the child with TB experienced stigma. The survey measured anticipated (21 items), enacted (23 items for PWTB and 24 items for caregivers), and internal (16 items) stigma (Appendix Table S1). Measurements were made using a five-level Likert scale (‘strongly agree’, ‘agree’, ‘neutral’, ‘disagree’, and ‘strongly disagree’). These included questions to ascertain how different stigma experiences affected each stage of the care cascade from care-seeking, to diagnosis, to treatment initiation, completion or cure. We designed a semi-structured in-depth interview guide (Appendix) to cover three key topics: *a) the person’s TB illness narrative, b) details about their experiences relating to anticipated stigma, enacted stigma and internal stigma, and c) recommendations for interventions to reduce stigma*. The PRAs pre-tested the interview guide and developed specific examples of (i) internal (ii) anticipated and (iii) enacted stigma based on personal experiences to illustrate these concepts, and to establish rapport and make participants feel at ease during interviews.

### Data Collection

Two PRAs (GM, PT) administered the survey in Khayelitsha, and one CHW administered the survey in Hammanskraal. Survey data were collected in the Research Electronic Data Capture (RedCap) system (23). Survey participants did not receive reimbursement, but were entered into a raffle to win a small prize (worth R500 ≈ $26). In-depth interviews, lasting 30–60 minutes, were conducted by trained interviewers (PRAs: GM and PT, with support from researcher NV) at either the participant’s home or their local health clinic, and audio recorded. Interviews were conducted either in-person or via telephone. Field notes were written following each interview. In-depth interview participants received reimbursement for their time and transport (R100 ≈ $5.25). Surveys and interviews were conducted in the participants preferred language (isiXhosa in Khayelitsha; Sotho in Hammanskraal).

### Analyses

Quantitative: Descriptive statistics were used to describe socio-demographic and clinical survey data. In univariate analyses, we compared variables using the Student’s t-test, and one-way analysis of variance. We reported the proportions of participants who selected agree or strongly agree to at least one or more than five survey items describing experiences of anticipated (11 items), internal (10 items), and enacted (13 items) stigma, along with the mean proportions (24). We stratified data by gender. We generated standardized stigma scores for each stigma domain by summing the results for each item out of 5 with higher scores indicating higher stigma (minimum score 1 and maximum score 5). Good internal reliability of scales was a priori defined as an alpha of ≥ 0.7. The stigma scale in this study was internally consistent: Cronbach’s alpha was 0.904 for anticipated stigma, 0.897 for enacted stigma, and 0.880 for internal stigma in those who experienced TB. Generalized linear regression models were used to investigate the determinants of anticipated, enacted, and internal stigma. We included variables with p ≤ 0.1 using the Kruskal-Wallis test for inclusion in the multivariate models. Beta-coefficients (β coef) were presented with a 95% confidence interval. A 2-tailed p-value < 0.05 was considered statistically significant. Statistical analyses were conducted using STATA 16 (StataCorp LP, College Station, TX.)

Qualitative: In-depth interview audio files were transcribed and translated into English using professional transcription services. Transcripts were imported into NVivo 12 software (Lumivero, Colorado, USA). Two coders (IF, RRN) performed open coding to inductively identify emergent themes related to stigma experiences (25). No new themes were derived after 25 interviews, achieving data saturation (26). We then applied deductive thematic analysis by categorising themes according to the Constraints, Actions, Risks or Desires (CARD) framework (27), which integrates data on individual and systemic factors in a single analytical frame to inform intervention design. We used the CARD framework to analyze the interaction of factors within the networked ecosystem in which PWTB and caregivers contextualize and interpret their perspectives and behaviours related to TB stigma.

Quantitative and qualitative data were analyzed separately, and we triangulated the findings as part of our integrated mixed methods analysis.

### Ethics

This study was approved by the Human Research Ethics Committee of the Faculty of Health Sciences at Stellenbosch University, the City of Cape Town and Tshwane, South Africa (N20/10/113). For the surveys, which were anonymous, verbal consent was obtained electronically on the survey administration device. For interviews, written informed consent was obtained.

## RESULTS

### Stigma survey results

#### Survey population characteristics.

Surveys were administered to 117 individuals, including 93 (79.5%) PWTB and 24 (20.5%) caregivers. The median age of PWTB was 40 years (IQR 33–49), approximately half were women (53%), and 55 (59%) PWTB reported that they were living with HIV (Table 1). The majority of PWTB had pulmonary TB (91%) and 9% had drug-resistant TB (DR-TB). Over one-third of PWTB reported prior TB (37%) (Table 1). Most caregivers were women (58%). Most children had pulmonary TB (22/24) and three children had DR-TB; fewer were living with HIV (13%) (Table 1).

##### At least one-third of PWTB and one-quarter of caregivers reported they or their children experienced anticipated, internal, or enacted stigma, of which anticipated stigma was most common.

The mean proportion of PWTB experiencing anticipated, internal, or enacted stigma was 58%, 35%, and 42% respectively (Table 2). Almost all PWTB reported experiencing some form of anticipated, internal, or enacted stigma, with 96% (89 of 93) reporting agree or strongly agree to at least one item measuring these forms of stigma. The mean proportion of caregivers reporting their child with TB experienced anticipated, internal, and enacted stigma was 36%, 25%, and 24% respectively (Table 2). While caregivers overall reported stigma less commonly than PWTB, the majority of caregivers reported some form of anticipated (96%), internal (100%), and enacted (79%) stigma, based on their agreement or strong agreement with at least one item measuring each of these forms of stigma. A high proportion of PWTB anticipated stigma from both communities and families (57% and 55%), whereas enacted stigma was more commonly experienced in communities than from families (45% and 30%). Caregivers reported similar frequencies of stigma experiences in community and family settings for anticipated (36% and 32%) and enacted (25% and 23%) stigma.

##### Anticipated, internal, and enacted stigma impacted engagement throughout the care cascade.

**Figure 1:**
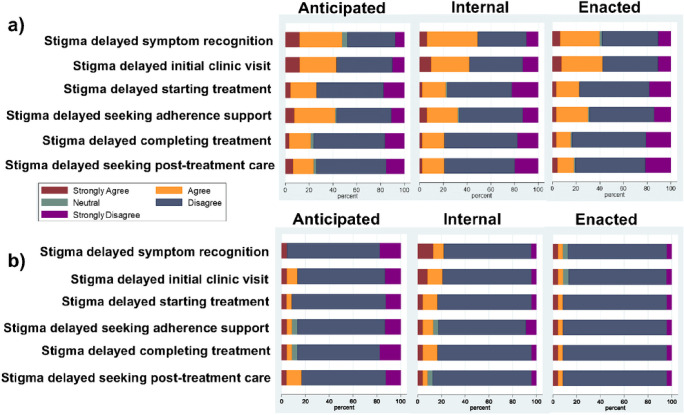
Impact of anticipated, internal and enacted stigma on care cascade engagement in a) PWTB and b) caregivers.

A high proportion (between 20–40%) of PWTB strongly agreed or agreed that experiences of all three types of stigma impacted care engagement at sequential steps of the cascade. Importantly experiences of stigma continued to be frequet after PWTB have already engaged with care, which presents an opportunity for care services to improve the care of existing patients (Figure 1a). Caregivers more often disagreed that stigma impacted care cascade engagement (Figure 1b).

###### Living in a rural area, compared to a peri-urban setting was associated with increased stigma.

Univariate linear regression analyses demonstrated increased scores for anticipated, internal, and enacted stigma experienced by PWTB in Hammanskraal and who reported prior TB (Table 3). Univariate analyses suggested extra-pulmonary TB was associated with lower internal and enacted stigma scores, and that drug-resistant TB was associated with lower internal stigma scores. Univariate analyses did not demonstrate differences between stigma scores reported by caregivers (Appendix Table S2). Gender and HIV were not associated with differences in stigma scores. We included age, location, prior TB history, TB type, and drug resistance pattern in the multivariate models. Only living in Hammanskraal (rural area) compared to Khayelitsha (peri-urban area) was associated with higher scores in the multivariate model: internal stigma (β coef 0.71; 95% CI: 0.40, 1.03; p<0.001), anticipated stigma (β coef 0.72; 95% CI: 0.46, 0.97; p<0.001) and enacted stigma (β coef 0.74; 95% CI: 0.50, 0.97; p<0.001) (Table 3).

###### Gender is associated with different experiences of each type of stigma.

While stigma measure scores did not differ when stratified by gender, examination of responses to individual items revealed differences (Table 4). Compared to men, women were more likely to report anticipated stigma related to fears about gossip or losing friends. Women were more likely to report internal stigma due to themselves feeling that they should be kept away from other people, whereas men were more likely to feel internal stigma due to the association of TB with behavioral risk factors (i.e., alcohol use or smoking). Similarly, women were more likely to report enacted stigma due to people taking badly about them, losing respect in the community, or being undermined by people due to having TB, whereas men were more likely to report enacted stigma due to the association others made with stigmatized behaviours such as drinking or smoking (Table 4).

### In-depth interview findings

We interviewed 21 PWTB (14 women, six men), eight of whom had DR-TB and five of whom had HIV. We interviewed four caregivers (four women who took care of three boys and one girl), one of whom had HIV. The majority of interview participants lived in Khayelitsha (22); three lived in Hammanskraal. Key findings are presented according to the domains of the CARD framework.

#### Constraints: Stigma, including intersectional stigma due to HIV and alcohol use, is a major barrier to TB care, which is often linked with other important socioeconomic barriers.

Participants reported internal stigma, linked in part to physical changes (i.e., weight loss) that signified disease to others. Internal stigma was tied to intersectional stigma related to behaviours commonly associated with TB such as smoking and alcohol use. HIV-related stigma was the most pervasive intersectional stigma, due to the assumption that PWTB must also have HIV. Anticipated stigma resulted from fears that friends or community members would see participants seeking TB care or find out they had TB. Enacted stigma included feeling abandoned by friends who stopped visiting after learning of their TB diagnosis and being discriminated against by health workers. In terms of gender-based differences in stigma, embarrassment or shame about having TB was described as a barrier to men seeking and remaining engaged with TB care whereas women appeared to be more impacted by anticipated or enacted stigma related to the effect of TB on their physical appearance.

“When a person is looking at you, it is as if they are feeling pity for you. They think they are better, that they will never be infected with TB.” (016, man, MDR-TB, Hammanskraal)

“There’s a person at the community who I don’t talk to, who said things about me when I went outside. “Look at her, she walks slowly”. (018, woman, MDR-TB, HIV, Khayelitsha)

Participants described various socio-economic constraints, sometimes linked to stigma, often with long-term ramifications, including the loss of employment due to being sick with TB. Food insecurity was common. Although some had received social benefits through disability grants via the South African Social Security Agency (SASSA) program (28), participants reported challenges obtaining these, and that disability grants funds were insufficient to achieve food security for them and their families.

“[My life] changed because I’m unemployed. I’m struggling in life because I must live with SASSA and I can’t do the things I used to do even now [post TB]. With this SASSA the food gets finished quickly because I must also feed other people.” (003, woman, MDR-TB, Khayelitsha)

#### Actions: Stigma negatively affected behaviours including unwillingness to disclose their TB diagnosis and fears about (re-)engaging in care, although examples of stigma resilience were apparent.

Internal stigma was a driver of delayed care seeking, with feelings of negative self-worth persisting beyond TB treatment completion. Anticipated stigma affected care engagement at multiple cascade steps, including delayed care seeking and loss to follow up. Anticipated stigma was also a major driver for participants actively deciding not to disclose their TB diagnosis, to friends, schoolmates, and even to close family members or partners. Men appeared to be less likely to disclose their TB diagnosis than women, leading to feelings of isolation and negatively impacting social relationships.

“He might end up not coming to clinic. When he witnesses those who are marginalised, he might end up being afraid of coming to clinic because he thinks he will be marginalised.” (012, man, Khayelitsha)

“I never told her [participant’s girlfriend] anything - even today. We even broke up because I was ashamed what I would say.” (019, man, recurrent TB, Khayelitsha)

Fears of anticipated stigma and experiences of enacted stigma from health workers affected participants’ decisions about seeking and/or remaining engaged in care. Participants described their vulnerability during the first weeks of TB treatment as being met with enacted stigma from health workers, which was compounded for PWTB who were re-engaging with care if they had previously been lost to follow-up. To avoid HIV intersectional stigma, some participants actively showed friends or family that their medications were for TB and not for HIV.

“I showed [my friends] that I take treatment for TB and it’s not for AIDS [laughs]. They were thinking that I am taking treatment for AIDS and hiding it from them. I took the treatment in front of them.” (002, woman, Khayelitsha).

Some participants described beliefs and attitudes that appeared to make them resilient to the different experiences of TB stigma. This resilience often appeared to arise from understanding and acknowledging the universality of the risk of TB infection, which resulted in those participants avoiding self-blame and expecting that they should not be treated differently (or worse) than people without TB.

“This is an illness. No one is supposed to be treated badly because a person didn’t ask to have TB. A person is supposed to be treated the same. Don’t look down on them.” (001, woman, Khayelitsha)

Participants who did disclose their TB status often mentioned practical reasons or benefits for doing so, including logistical and emotional support, which served as strong enablers to treatment completion. Others described empowerment due to feeling like a hero after surviving TB treatment and not feeling ashamed about their physical impairment during their illness.

“I never felt bad that people were seeing me in a wheelchair. Now they see me walk. I take myself as a hero.” (004, man, MDR-TB, recurrent TB, Khayelitsha)

#### Risks: TB diagnosis was associated with negative mental health impacts that led to care disengagement, and risk perceptions related to infectiousness from friends and community members.

Being diagnosed with TB and resulting experiences of stigma often had a negative impact on PWTB mental health, with several reporting depression explicitly. Participants mentioned being in denial about their symptoms or illness. Several reported self-isolation, which was linked to internal stigma. This led to loneliness and poor psychological well-being, and contributed to care disengagement.

“ I didn’t even know it was depression because I didn’t even want to cooperate.” (024, woman, XDR-TB, Khayelitsha).

Internal stigma also arose due to fears about the risk of severe illness associated with TB with significant impacts on participants’ daily lives including feelings of hopelessness; this was compounded in people with DR-TB. At least one participant linked their fears to the pervasive belief among community members that the inevitable prognosis for TB is death. When this belief was re-affirmed through news of another TB patient dying, they became increasingly fearful for their life.

Anticipated and enacted stigma, driven by fears about infectiousness, fuelled concerns about the risks posed to others. This resulted in PWTB isolating themselves from others and others excluding them. Participants reported that misperceptions about them continuing to be infectious despite being on treatment acted as a stigma driver and led to further isolation of PWTB in homes and communities.

“[On why persons with TB are treated differently] They are afraid to be infected by a person who has TB. But they don’t have the knowledge about TB, because when you are taking the treatment, it’s not contagious.” ( 020, woman, Khayelitsha)

#### Desires: participants suggested that internal and anticipated stigma could be mitigated by offering counselling for PWTB and their families, and that anticipated and enacted stigma could be reduced by providing health workers with stigma sensitivity training, and community-wide education about stigma.

Desires related to reducing stigma and improving the well-being of PWTB and TB-affected communities fell into three main categories: counselling for PWTB and their families, stigma reduction training for health workers, and education for communities to close knowledge gaps and decrease stigma. Participants often mentioned the lack of counselling during TB diagnosis and/or TB treatment as an important barrier to delivering high-quality, person-centred care. They recommended peer counselling and noted that TB survivors could offer unique support to people undergoing TB treatment, based on their lived experience. Participants described current interactions with the health staff as limited to brief treatment information sessions (if they were received) and noted the lack of psychological support offered.

“I never received any counselling. They didn’t explain it to me; they just gave me pills only.” (005, woman, MDR-TB, Khayelitsha)

Efforts to reduce enacted stigma towards PWTB by health workers were thought to be particularly important when addressing people who are lost to follow- up (still commonly referred to as ‘defaulters’ by PWTB and health workers), with the goal of ensuring people are not judged or discriminated against. Participants noted that training, informed by patient perspectives, could improve health workers’ understanding of the challenges experienced by PWTB, enabling them to provide person-centred care.

“And at the hospital, a person is shouted at by the nurses that they don’t take their pills: “that’s why you default”. That name came to me, that I defaulted.” (018, woman, MDR-TB, Khayelitsha)

Participants mentioned the lack of community TB knowledge and awareness as important drivers of stigma and poor care engagement. Clinics or community venues, including schools, were mentioned as potential locations where TB-related education could take place. Participants emphasized the importance of ensuring communities have accurate information to understand how TB can affect anyone, how TB is spread, and how long people with TB remain infectious to others after treatment.

“[I recommend] education in the community so that they know if a person has been diagnosed with TB, how can they handle that person and how do they give them support.” (021, woman, recurrent TB, Khayelitsha)

### Triangulation of quantitative and qualitative findings

Interviews provided explanatory data on the negative effects of all three types of stigma experiences as measured in the quantitative surveys. Participants described how stigma, emanating from the community, resulted in anticipated and internal stigma and hesitation to seek care. They also described how anticipated and enacted stigma driven by fears of infectiousness, and resulting discriminatory attitudes by community members and health workers, affected retention in care. Some of the gender-based differences in stigma experiences noted in the survey analyses were corroborated by interview findings. For example, changes in appearance and status affect women’s psychological well-being but may not prevent them engaging with care. In comparison, discrimination due to intersectional stigma associated with alcohol use and smoking led to men feeling ashamed and less likely to engage in care. While HIV was not associated with higher stigma scores, interview participants often mentioned HIV intersectional stigma and how it affects PWTB both with and without HIV. The low number of interview participants from Hammanskraal limited exploration of factors that may explain why stigma scores were higher among survey participants in that setting.

### Example stigma intervention components based on mixed methods stigma assessment findings

Table 5 uses the CARD framework to organize and summarize the findings of our mixed methods stigma assessments. We report illustrative quotations and insights for stigma intervention design, including example stigma intervention components that were identified by the research team, including the PRAs.

## Discussion

Community-based surveys in two settings in South Africa reveal that more than one-third of PWTB and more than one-quarter of caregivers of children with TB reported anticipated, internal, or enacted TB stigma. Stigma experiences negatively affected care engagement throughout the care cascade. Stigma scores were higher in the rural community compared to the peri-urban community. Analysis of in-depth interviews using the CARD framework identified constraints, actions, risks, and desires that inform stigma intervention design. Anticipated, internal, and enacted stigma, including HIV intersectional stigma, act as key constraints, often compounded by socioeconomic barriers, to affect care engagement. Specifically, stigma experiences negatively affected actions such as decisions about care seeking, engagement and disclosure, although some participants displayed beliefs and attitudes that fostered stigma resilience. Risks linked to stigma included loss to follow-up and long-term impacts on mental health and well-being. Participants desired counselling and educational interventions to reduce stigma in families, communities, and health systems.

Triangulation of quantitative and qualitative data helps to understand how women and men appear to experience stigma differently. Interventions should be tailored to address drivers of anticipated and enacted stigma such as the negative impacts on women that may lead to social isolation versus effects of intersectional stigma on men that may lead to social exclusion. Our study aligns with others (29) to emphasize the need for multi-component interventions that address different stigma experiences, such as counselling for people and families affected by TB, and health worker training and education for communities to reduce stigma drivers.

Our findings illuminate how stigma affects decisions and behaviours surrounding care engagement and disclosure. They further highlight gaps in the current approach to counselling, which typically focuses on treatment adherence and is not a patient-centred approach to providing support during TB care and treatment. Effective interventions need to address the drivers of stigma such as blame and fears of transmission, along with intersectional stigma due to HIV, alcohol and substance use, which we and others suggest is experienced differently by men and women (29). By intervening on the drivers of stigma, the stigmatisation process can be interrupted prior to its occurrence, rather than post-occurrence when interventions serve to mitigate the effects of stigma (30).

Stigma resilience appeared to be associated with PWTB feeling able to disclose their illness to their families, which then opened up access to mental and tangible resources. This may explain some of the gender-based differences in outcomes if men are less likely to disclose and seek support. PWTB who understood that TB can affect anyone and did not link the disease to their self-worth appeared to be more resilient to stigma, highlighting potential messaging for counselling interventions. While empowering PWTB through counselling can help them mitigate internal and anticipated stigma, interviews emphasized the parallel need to address discrimination and enacted stigma by health workers and community members. Our data support systematic and scoping review recommendations that suggest internal and anticipated stigma may be addressed effectively with interventions targeted at individuals using counselling or support groups, whereas enacted stigma may be better addressed with information-based interventions targeted at the organisational or community level (31, 32).

We found high levels of stigma that continue to occur after people engage with TB care services, underscoring the potential impact of stigma interventions on reducing high rates of loss-to-follow up post TB diagnosis. This is important since this target population has already engaged with the health system in contrast to those who remain undiagnosed (and may be perceived as being harder to reach). Nonetheless, another cross-sectional study conducted in South Africa, demonstrated higher stigma in presumptive patients compared to those post-diagnosis (33), which suggests the importance of dispelling stigma through community focused interventions (29). Results from a large mixed-methods study to understand the impact of TB-related stigma, intersectional stigma, and social determinants of health in the Eastern Cape province of South Africa will further advance understanding how stigma manifests across the cascade (34).

Our interviews highlighted how stigma affects mental health, including experiences of overt depression and the long-term impact of TB on well-being. Unfortunately, neither stigma nor mental health are well-addressed by current models of care. Our findings, and those of others (35), underscore the importance of integrated mental health and TB services. Implementation research is needed to evaluate the integration of evidence-based interventions for co-morbid depression among PWTB.

Social movements such as for disability rights highlight the importance of community engaged approaches, with a central tenet of ‘Nothing about us, without us’ (36). Our study demonstrates how insights from community-engaged research that is implemented in partnership with TB survivors can inform intervention design, including consideration of how context affects stigma. Increased TB stigma in rural areas compared to urban areas has been previously reported (33, 37), although other studies have shown higher or similar levels of stigma in urban areas (37, 38). We note that differences in the cultural groups and norms of our two study communities likely contribute to differences in experiences and impacts of stigma. Such differences merit further evaluation.

In contrast to TB, efforts to reduce HIV-related stigma have been a key component of the global HIV response. Adapting strategies used to address HIV stigma, including the prioritization of rights-based approaches and empowerment, can help to change narratives surrounding TB. This includes eliminating criminalizing and discriminatory terms such as suspect or defaulter that drive stigma (14), and considering personal rather than only public health motivation to facilitate disclosure conversations (39). Given stigma’s impact on TB care outcomes as well as longer-term impacts on people’s well-being, we recommend that TB-related stigma should be evaluated and measured as a primary indicator of quality-of-life rather than merely as a programmatic or medicalized indicator of the TB cascade (8). It is important to note here that TB stigma is a social determinant of health (40). As such, eliminating stigma differs from other interventions such as implementing a new diagnostic test or regimen, as it is intrinsically linked to other complex issues including poverty and racism (41–43).

Strengths of this study include the measurement of different stigma experiences using a validated stigma assessment tool (21) in two geographically distant communities with different cultural characteristics. The use of mixed methods to enable triangulation of quantitative and qualitative data enhances the validity and credibility of our findings. Employing the CARD framework enabled us to apply our findings to directly inform intervention design. The use of a community-based participatory research approach that included TB survivors as PRAs fosters research equity, maximizes the engagement of affected communities, and ensures relevance and implementation of the research data (44). Another strength is mutual ownership and leadership of this project by members of a TB advocacy organization. This allows for wider dissemination and use of findings to guide effective public health and advocacy messaging, such as emphasizing the universality of TB risk to nurture stigma resilience.

Study limitations include lower numbers of survey participants than initially planned due to the impact of the COVID-19 pandemic on study staffing and enrollment. COVID-19 also prevented us from interviewing more participants, especially caregivers, and doing so in Hammanskraal. This limited our ability to explore contextual differences in stigma between our study sites, the effects of stigma on caregiving and its impact on their behaviors and caregiving. Potential selection bias due to the sampling facilitated by community health worker referrals was possible for both qualitative and quantitative data collection but it is uncertain whether this could have led to people who experienced higher versus lower stigma being included. Given that the majority of our survey and IDI participants were women, we acknowledge selection bias with respect to gender; men represent 62% of PWTB in South Africa (45). This likely limits our ability to further delve into gender-based dimensions of stigma.

## Conclusion

Multi-component and multi-level approaches are needed to address the different experiences of stigma that additively and synergistically impede engagement in TB services. Interventions must incorporate contextual differences that may arise due to gender or setting, address HIV and other intersectional stigmas, and incorporate messaging from survivors to foster stigma resilience. Given its impact on care outcomes and long-term well-being, TB stigma should be measured as an outcome in itself, rather than merely considered as an intermediate step to improving programmatic outcomes. Community-engaged, evidence-based approaches to address stigma must be a key priority for TB elimination efforts. Such efforts require multisectoral collaboration to address broader issues including social protection, health system strengthening, and community empowerment.

## Figures and Tables

**Table 1:** Study participant characteristics for PWTB and caregivers of children with TB.

	People with TB (n=93)	Caregivers (n=24)	Total (n=117)

*Demographic Variables*			

**Median Age (years, IQR)**	40 (33–49)	*	-

**Gender, No (%)**	49 (53)	14 (58)	63 (54)
Woman	44 (47)	10 (42)	54 (46)
Man			

**Language, No (%)**	10 (11)	4 (17)	14 (12)
English	37 (40)	20 (83)	57 (49)
Xhosa	45 (48)	0 (0)	45 (38)
Sotho	1 (1)	0 (0)	1 (1)
Missing			

**Location, No (%)**	47 (51)	23 (96)	71 (61)
Khayelitsha	45 (49)	0 (0)	45 (38)
Hammanskraal	1 (1)	1 (4)	2 (1)
Missing			

***Clinical Variables***			

**HIV, No (%)**	55 (59)	3 (13)	58 (50)
Yes	18 (19)	8 (33)	26 (22)
No	9 (10)	2 (8)	11 (9)
Refused	11 (12)	11 (46)	22 (19)
Missing			

**TB Type, No (%)**	85 (91)	22 (92)	107 (91)
Pulmonary	8 (9)	2 (8)	10 (9)
Extra-pulmonary			

**Drug Resistance Type, No (%)**	85 (91)	21 (87)	106 (91)
Drug Sensitive	8 (9)	3 (13)	11 (9)
MDR or XDR			

**Previous TB, No (%)**	34 (37)	-	-
Yes	59 (63)		
No			

Abbreviations: IQR= Interquartile Range, SD= Standard Deviation, MDR=Multidrug Resistant, XDR= Extensively Drug Resistant

* Age was often inconsistently recorded as the age of the caregiver instead of the child thus not reported here.

**Table 2:** Experiences of stigma and settings where these occurred.

	PWTB (n=93)	Caregivers (n=24)	Total (n=117)

***Average proportion of participants experiencing these types of stigma***			

**Anticipated stigma, n(%)**	54 (58)	9 (36)	63 (54)

**Internal stigma, n(%)**	33 (35)	6 (25)	39 (31)

**Enacted stigma, n(%)**	39 (42)	6 (24)	45 (38)

***At least 1 survey item with an agree/strongly agree response***			

**Anticipated stigma (11 items), n(%)**	89 (96)	23 (96)	112 (96)

**Internal stigma (10 items), n(%)**	89 (96)	24 (100)	113 (97)

**Enacted stigma (13 items), n(%)**	89 (96)	19 (79)	108 (92)

***At least 5 items with an agree/strongly agree response***			

**Anticipated stigma (11 items), n(%)**	67 (72)	12 (50)	79 (68)

**Internal stigma (10 items), n(%)**	40 (43)	11 (46)	51 (44)

**Enacted stigma (13 items), n(%)**	52 (56)	9 (38)	61 (52)

***Settings of stigma experiences***			

Anticipated stigma-Community	53 (57)	9 (36)	62 (53)
Yes	29(31)	13 (55)	42 (36)
No	51 (55)	8 (32)	59 (50)
Anticipated stigma-Family	40 (43)	16 (67)	56 (48)
Yes			
No			

Enacted stigma-Community	42 (45)	6 (25)	48 (41)
Yes	39 (42)	15 (62)	54 (46)
No	28 (30)	6 (23)	34 (29)
Enacted stigma-Family	62 (67)	18 (73)	80 (68)
Yes			
No			

* Proportion of participants who experienced stigma based on mean of total participants who reported strongly agree or agree with any of the items for anticipated, internal, or enacted stigma.

**Table 3:** Factors associated with stigma experienced by people with TB.

	Anticipated Stigma				Internal Stigma				External Stigma			

Variable	Crude β-coef (95% CI)	p-value	Adjusted β-coef (95% CI)	p-value	Crude β-coef (95% CI)	p-value	Adjusted β-coef (95% CI)	p-value	Crude β-coef (95% CI)	p-value	Adjusted β-coef (95% CI)	p-value

**Age Categories, No (%) in years**												
<30	*Reference*	-	*Reference*	-	*Reference*	-	*Reference*	-	*Reference*	-	*Reference*	-
31–40	**0.41 (0.05, 0.78)**	**0.027**	−0.03 (−0.38, 0.054 0.31)	0.860	0.32 (−0.14, 0.77)	0.168	−0.22 (−0.64, 0.20)	0.308	0.31 (−0.03, 0.66)	0.076	−0.18 (−0.49, 0.14)	0.266
41–50	0.38 (−0.01, 0.76)	0.054	−0.01 (−0.37, 0.35)	0.960	0.15 (−0.33, 0.62)	0.543	−0.28 (−0.72, 0.15)	0.197	0.24 (−0.13, 0.60)	0.208	−0.20 (−0.52, 0.13)	0.233
51–60	0.06 (−0.39, 0.51)	0.798	−0.01 (−0.41, 0.39)	0.958	0.03 (−0.53, 0.59)	0.916	−0.10 (−0.59, 0.39)	0.683	−0.11 (−0.55, 0.32)	0.602	−0.23 (−0.60, 0.13)	0.201
>60	−0.27 (−0.92, 0.37)	0.404	−0.15 (−0.77, 0.47)	0.627	−0.24 (−1.04, 0.57)	0.561	−0.15 (−0.91, 0.61)	0.690	−0.25 (−0.87, 0.37)	0.429	−0.19 (−0.66, 0.38)	0.513

**Gender, No (%)**												
Woman	*Reference*	-	-	-	*Reference*	-	-	-	*Reference*	-	-	-
Man	−0.03 (−0.27, 0.22)	0.837			−0.09 (−0.30, 0.12)	0.388			−0.18 (−0.41, 0.05)	0.121		

HIV, No (%)*												
Yes	*Reference*	-	-	-	Reference	-	-	-	*Reference*	-	-	-
No	−0.09 (−0.28, 0.10)	0.354			−0.08 (−0.37, 0.22)	0.609			−0.15 (−0.33, 0.04)	0.113		

**History of Previous TB, No (%)**												
Yes	*Reference*	-	*Reference*	-	*Reference*	-	*Reference*	-	*Reference*	-	*Reference*	-
No	**0.31 (0.06, 0.55)**	**0.016**	0.05 (−0.18, 0.28)	0.680	**0.53 (0.25, 0.81)**	**<0.001**	0.27 (−0.02, 0.55)	0.067	**0.33 (0.10, 0.56)**	**0.006**	0.11 (−0.10, 0.33)	0.288

**TB Type, No (%)**												
Pulmonary	*Reference*	-	*Reference*	-	*Reference*	-	*Reference*	-	*Reference*	-	*Reference*	-
Extrapulmonary	−0.33 (−0.77, 0.10)	0.128	0.09 (−0.30, 0.48)	0.647	**−0.55 (−1.06, −0.04)**	**0.035**	−0.01 (−0.47, 0.49)	0.965	**−0.45 (−0.86, −0.05)**	**0.030**	−0.06 (−0.42, 0.30)	0.734

**Drug Resistance, No (%)**												
Drug Sensitive	*Reference*	-	*Reference*	-	*Reference*	-	*Reference*	-	*Reference*	-	*Reference*	-
DR-TB	−0.43 (−0.85, 0.004)	0.052	−0.07 (−0.47, 0.33)	0.736	**−0.81 (−1.3, −0.32)**	**0.002**	−0.33 (−0.82, 0.16)	0.184	−0.38 (−0.79, 0.03)	0.068	0.09 (−0.27, 0.46)	0.620

**Location, No (%)**												
Khayelitsha	*Reference*	-	*Reference*	-	*Reference*	-	*Reference*	-	*Reference*	-	*Reference*	-
Hammanskraal	**0.73 (0.53, 0.92)**	**<0.001 0.72**	**(0.46, 0.97)**	**<0.001**	**0.77 (0.52, 1.01)**	**<0.001**	**0.71 (0.40, 1.03)**	**<0.001**	**0.73 (0.56, 0.91)**	**<0.001**	**0.74 (0.50, 0.97)**	**<0.00**

**Table 4.** Stigma survey items for which responses differed between women and men with TB.

Survey Statement	Mean of Agree Responses (0–1): Women	Mean of Agree Responses (0–1): Men	P-value
**Anticipated Stigma**			
I have worried that people might talk badly/gossiped about me because I had TB.	0.429	0.205	0.022
**Internal Stigma**			
I have felt that having TB means I should be kept away from other people (alone)	0.469	0.250	0.029
I probably got TB because I smoke, drink, or do other careless things	0.265	0.500	0.020
**External Stigma**			
People have talked badly/gossiped about me because I had TB	0.796	0.591	0.032
I have lost respect/standing in the community because I had TB	0.694	0.341	0.001
I have been undermined by people because I had TB	0.633	0.364	0.010
Someone has told me that I have TB because I smoke, drink, or do things that other people might think of as ‘careless’	0.204	0.409	0.032

**Table 5.** Use of the CARD analytical framework to design stigma interventions informed by community-based stigma assessments.

CARD DOMAINS and Themes	Illustrative quotations	Researcher recommendations for applying components to a stigma intervention

**CONSTRAINTS** - Internal stigma - Anticipated stigma	*“I told myself that people who can get TB are smokers, people who work in cold places. I never thought that I would get TB.”*	Individual counselling for all TB patients is needed, including messaging that TB can happen to anyone and should not be seen as an indictment of someone’s worthiness, and discussion about how to address anticipated and external stigma that may arise.
- External stigma	*“What is so and so going to say when I'm on the side of people with TB, who are taking the TB treatment?”*	
	*“There were no people coming to my house when I was sick”*	

**CONSTRAINTS** - Intersectional stigma: HIV, alcohol use, gender	*“If you lose weight then they assume that you have HIV They would say you might have HIV and you should go and get tested.”*	Integrated TB/HIV stigma counselling and targeted education campaigns can educate communities on risk factors for TB and reduce intersectional stigma.
	*“He was drinking alcohol and he died.”*	Use of risk screening strategies can help identify people with alcohol or substance use at risk of poor outcomes, alongside messaging that anyone can develop TB.
		Gender-tailored care engagement approaches should address differences in stigma for men and women.
	*“Us man people, we are worse. We are embarrassed to go to the clinic and take treatment”*	

**CONSTRAINTS** - Socio-economic barriers and exacerbation of these due to TB	*“I struggled when it comes to food and I am still struggling now because I am not working.”*	Interventions should incorporate components such as food or transport vouchers.
	*“I don’t have money, I don’t have anything to eat. If I take these pills, they make me sick”.*	Information and assistance with obtaining social support should be available to TB patients.

**ACTIONS** - Disclosure decisions varied depending on anticipation and experiences of enacted stigma	*“[On the topic of telling friends about their TB] I knew that they were going to gossip about me.”*	Interventions should include members of a PWTB’s social network, such as family-centered counselling (for example family workshops and home visits to strengthen individual support networks).
	*“No! I never had any problem at all with telling them, I was free, free, free, they also know, they used to help me, my family encouraged me”.*	

**ACTIONS** - Stigma resilience demonstrated by understanding TB can affect anyone	*“I didn’t fear anything when it came to that [decision to seek healthcare], I just thought everyone can be infected by TB.”*	Education on universality of risk to TB infection can support de-linking TB infection with blame for persons infected.
	*“I was telling her that she [child with TB] is not the only one that has TB. There are a lot of people that have TB.”*	Support groups among PWTB can facilitate co-learning from disclosure experiences.

**RISKS** - Depression - Care disengagement more likely due to impacts on mental health	*“I had nightmares. Sometimes I had a dream of being buried. Maybe if I knew how depression starts, maybe I would say it was starting because there was a time where I didn’t want to be around people, I wanted to be alone. And sometimes, I didn’t even take those pills.”* *“Once a person has started treatment, we go around talking about them, saying that "So and so has TB”. This person is even afraid to leave the house, to go to the clinic.”*	Support groups among PWTB can facilitate co-learning and knowledge sharing on coping mechanisms to mitigate the risk of depression.
		Integration of screening and referral for mental health conditions into routine TB-care can further ensure PWTB are supported.

**RISKS** - Disease severity	*’People don’t have hope. When people talk about TB, they would say that person will die or say a person won’t survive. When I heard a certain person has died, and they had TB, I had that fear within me that “Wow! I also have TB”*	Counselling mechanisms including support groups and connection with TB survivors can help ensure people with TB are better informed on the outcomes of TB treatment and survival rates.

**RISKS** - Fear of infectiousness and transmission	*“People who were my friends, who I used to help, isolated themselves from me.”* *“I think he [child with TB] was also scared to go play with them since people say that TB is infectious.”*	Family workshops to provide education at the household level and community education interventions can support better understanding at the interpersonal and community level on infectiousness and transmission.

**DESIRES** - Counselling, including specific role for peer counsellors	*“I would say that they [counsellors] are needed, but should be someone who has experience of what you are dealing with, who has felt the pain you would be feeling like the side effects.”*	Counselling mechanisms including support groups and connection with TB survivors can help to ensure that people with TB feel supported as they undergo treatment.

**DESIRES** - Education for health workers and communities	*I said to the doctor “You educated people have a name saying ‘default’. You’re mistaken. I didn’t default, I’m just not well”.* *“I listen a lot to the things being taught at the clinics. We could have a venue where people will be invited to be taught about TB.”*	Educational campaigns can reduce mis-information and associated stigma, for example, targeted education and training for health workers or community campaigns can reduce stigma.

## Abbreviations

CHW: Community Health Worker
CARD: Constraints, Actions, Risks or Desires
DR-TB: Drug-Resistant TB
HIV: Human Immunodeficiency Virus
MDR-TB: Multidrug-resistant TB
PWTB: People with TB
PRA: Peer Research Associate
SASSA: South African Social Security Agency
TB: Tuberculosis
WHO: World Health Organization
XDR-TB: Extensively drug-resistant TB
